# Cerebral and Peripheral Changes Occurring in Nitric Oxide (NO) Synthesis in a Rat Model of Sleeping Sickness: Identification of Brain iNOS Expressing Cells

**DOI:** 10.1371/journal.pone.0009211

**Published:** 2010-02-16

**Authors:** Donia Amrouni, Sabine Gautier-Sauvigné, Anne Meiller, Philippe Vincendeau, Bernard Bouteille, Alain Buguet, Raymond Cespuglio

**Affiliations:** 1 University of Lyon, Faculty of Medicine, EA 4170 Laboratory of Free Radicals, Energy Substrates and Cerebral Physiopathology, & Neurochem platform, Lyon, France; 2 University of Bordeaux 2, EA 3677 Laboratory of Parasitology, Bordeaux, France; 3 University of Limoges, EA 3174 Laboratory of Tropical and Compared Neuroepidemiology & IFR 145 GEIST, Faculty of Medicine, Limoges, France; University of São Paulo, Brazil

## Abstract

**Background:**

The implication of nitric oxide (NO) in the development of human African trypanosomiasis (HAT) using an animal model, was examined. The manner by which the trypanocidal activity of NO is impaired in the periphery and in the brain of rats infected with *Trypanosoma brucei brucei* (*T. b. brucei*) was analyzed through: (i) the changes occurring in NO concentration in both peripheral (blood) and cerebral compartments; (ii) the activity of nNOS and iNOS enzymes; (iii) identification of the brain cell types in which the NO-pathways are particularly active during the time-course of the infection.

**Methodology/Principal Findings:**

NO concentration (direct measures by voltammetry) was determined in central (brain) and peripheral (blood) compartments in healthy and infected animals at various days post-infection: D5, D10, D16 and D22. Opposite changes were observed in the two compartments. NO production increased in the brain (hypothalamus) from D10 (+32%) to D16 (+71%), but decreased in the blood from D10 (−22%) to D16 (−46%) and D22 (−60%). In parallel with NO measures, cerebral iNOS activity increased and peaked significantly at D16 (up to +700%). However, nNOS activity did not vary. Immunohistochemical staining confirmed iNOS activation in several brain regions, particularly in the hypothalamus. In peritoneal macrophages, iNOS activity decreased from D10 (−83%) to D16 (−65%) and D22 (−74%) similarly to circulating NO.

**Conclusion/Significance:**

The NO changes observed in our rat model were dependent on iNOS activity in both peripheral and central compartments. In the periphery, the NO production decrease may reflect an arginase-mediated synthesis of polyamines necessary to trypanosome growth. In the brain, the increased NO concentration may result from an enhanced activity of iNOS present in neurons and glial cells. It may be regarded as a marker of deleterious inflammatory reactions.

## Introduction

Human African trypanosomiasis (HAT), or sleeping sickness, is a reemerging parasitic disease caused by protozoan parasites belonging to the genus *Trypanosoma*. The disease, always fatal if untreated, is transmitted by the bite of an infected insect vector, the tsetse fly. Two sub-species of *Trypanosoma* infective to humans are present in 36 Sub-Saharan African countries. Two clinical entities exist due to the geographic distribution of *Trypanosoma* sub-species. *Trypanosoma brucei (T. b.) gambiense*, found in West and Central Africa, causes a chronic infection with slowly evolving symptoms (months to years) whereas *T. b. rhodesiense*, found in East Africa, is responsible for an acute sickness (weeks to months). In both clinical entities, two distinct stages occur successively during the course of the disease. The early stage, or hemolymphatic stage 1, corresponds to parasite proliferation in blood and lymphatic systems, the parasites invading progressively peripheral vital organs. The late or meningoencephalitic stage 2 is characterized by the crossing of the blood-brain barrier (BBB) with the invasion of the central nervous system (CNS) by the parasites [1]. Although they may be observed throughout the course of the disease, neurological signs are most often described during stage 2, with a wide panel of sensory, motor and psychiatric disturbances, and the development of disruptions of the sleep-wake cycle, a most characteristic sign that gave its name to the illness [2]. A similar time course with two successive clinical stages together with sleep-wake cycle disturbances has been described in animal models infected by *T. b. brucei* [3].

In recent years, immune and neurological reactions to trypanosomal infection have been related to a gaseous signaling molecule, nitric oxide (NO) that represents an essential messenger between trypanosomes and the immune and nervous systems [4]. Nitric oxide is synthesized from the amino acid L-arginine by the enzymatic family of NO synthases (NOS) that consist of three major isoforms: neuronal NOS (nNOS, type 1), inducible/or macrophagic NOS (iNOS, type 2) and endothelial NOS (eNOS, type 3) [5]. The NO generated by iNOS is a key cytotoxic weapon used by the innate response for the destruction of trypanosomes [6] through the combination of NO and oxygen reactive species. However, proliferation of trypanosomes in the vicinity of peripheral macrophages in infected mice suggested the existence of a reduced efficiency of NO-dependent cytotoxicity. The competition between arginase and iNOS for their common substrate, L-arginine, reduces NO production and favors polyamine synthesis, required for trypanosome development [7]. Using specific voltammetric sensors [8] *in vivo*, our team has previously reported that NO is decreased in the blood of rats [9] and mice infected by *T. b. brucei* [10] and in humans infected with *T. b. gambiense* [10].

In the CNS, studies using a combination of polysomnographic recordings and micropharmacological treatments employing NO synthesis inhibitors or NO donors, have shown that NO is clearly involved in the modulation of the sleep-wake states [11]. In the experimental rat model of HAT, the hypothalamus, possessing a neuronal network involved in the regulation of the sleep-wake states [12], seems to be particularly vulnerable. Pro-inflammatory cytokines (IL-1, TNF-α) and activation of microglia were detected in periventricular cavities and most importantly in the hypothalamic area [1]. Such a pathological evolution might be at the origin of circadian rhythm and neurological disturbances. In our previous voltammetric studies, we demonstrated that NO concentration is increased in the brain cortex of rats and mice [9], [10]. This up-regulation of the NO pathways remains, however, to be further investigated, notably in the hypothalamus since the respective role played by the different NOS isoforms in the NO enhancement remains to be defined more precisely.

The present study, using an experimental rat model of human sleeping sickness (HAT), was designed to evaluate the changes occurring in NO production throughout the time-course of *T. b. brucei* infection. We chose to compare brain tissue and blood because these compartments are also those currently investigated in HAT: cerebrospinal fluid (CSF) for the brain tissue and blood for periphery. We emphasized the fact that the impairments taking place in these compartments reflect the two stages of the disease. To achieve this objective, the first step was to confirm that the experimental disease evolved in two successive stages by measuring body weight and the parasitic charge in the peripheral (blood) and cerebral (CSF) compartments. Afterwards, we focused on the original part of our approach, i.e., the changes occurring in NO concentration in the two compartments, together with an evaluation of enzymatic activity (nNOS and iNOS) and the identification of the cell types in which the NO-pathways are particularly active during the course of the infection.

## Materials and Methods

### Animals

Male rats (Wistar strain, 200–220 g, Janvier breeding, Le Genest Saint Isle, France), housed in individual cages, were kept under standard laboratory conditions (12/12h light/dark cycle, light-on at 5.00 a.m.; 22±1°C; water and food *ad libitum*). After 8 days of adaptation to the experimental conditions, the animals were randomly divided in two groups, a control (24 animals) and an infected group (24 animals). All experiments were performed according to the guidelines of the French Agriculture Ministry (N°: 03-505).

### Infection, Body Weight Follow-Up, Parasitemia and Cerebrospinal Fluid (CSF) Trypanosome Count

Rats were infected by intraperitoneal (i.p.) injection of 3600 parasites (*T. b. brucei* AnTat 1.1E clone, provided by Institute of Tropical Medicine, Antwerp, Belgium) per animal. Prior to the injection, mobility of trypanosomes was observed under microscope. In parallel, control animals received an i.p. injection of the same volume of saline solution. Body weight and blood parasite counts were monitored every two days, while CSF parasite counts were performed every four to five days. CSF samples were obtained by *cisterna magna* puncture from anesthetized (chloral hydrate, 400 mg/kg) rats kept in a stereotaxic frame with body temperature maintained at 37°C by a homoeothermic blanket (Harvard apparatus, Les Ulis, France). After reclining the neck muscles from the midline, CSF was collected with a syringe guided in the *cisterna magna* by way of a stereotaxic holder. Before reaching the *cisterna magna*, the needle was inserted gently through the meninges.

### Dissection and Tissue Preparation

The rats were sacrificed by decapitation at days 5, 10, 16 or 22 post-infection (D5, D10, D16 and D22). This timing was chosen from previously published data [3] as representing the middle of the first hemolymphatic stage (D5), the limit between stages 1 and 2 (D10), the well-established neurological stage 2 (D16) and the *pre-mortem* condition (D22). Blood was sampled by direct heart puncture. Macrophages were collected by washing the peritoneal cavity with a saline solution (NaCl 0.9%) containing heparin (0.4%) and polymyxine B (1 mg×L^−1^). Brains were quickly removed, briefly rinsed in ice-cold Tris-EDTA buffer (50 mM, pH 7.4) and dissected to collect hypothalamic and thalamic structures.

#### Protein extracts from brain tissue

After dissection, hypothalamic and thalamic samples were homogenized by sonication in 1 mL of ice-cold Tris-EDTA buffer (50 mM, pH 7.4) containing EDTA (1 mM), dithiothreitol (1 mM) and protease inhibitor cocktail (Sigma-Aldrich, St Quentin-Fallavier, France,). Homogenates were centrifuged at 15,000 g for 45 min at 4°C and aliquots of the supernatants were collected and stored at −80°C until biochemical assay. Protein concentration in supernatant fractions was determined using Bradford's method [13].

#### Protein extracts from peritoneal macrophages

The collected peritoneal cells (in suspensions) were centrifuged at 1,000 g (22°C, 5 min), and placed in MEM containing 2% fetal calf serum and 1% penicillin-streptomycin. Afterwards, cells (1×10^6^/2mL/well) were layered on 6-well plates and maintained at 37°C under 5% CO_2_ atmosphere during 2 hours. Non-adherent cells were removed by three successive washings with phosphate buffer saline (PBS, pH 7.4) and adherent cells (macrophages) were used for protein extraction as described above for brain extracts. Protein concentration in supernatant fractions was determined using Bradford's method [13].

### NO Measurements

#### *In vivo* NO measurements in the brain

Detection of NO was achieved using a NO sensor in combination with differential normal pulse voltammetry (DNPV) as previously described [8]. NO measurements were performed under chloral hydrate anesthesia (400 mg/kg i.p. injection). Animals were kept on a stereotaxic frame with body temperature maintained at 37°C by a homoeothermic blanket (Harvard apparatus, Les Ulis, France). The superficial cutaneous and muscular layers were resected and bone trepanation was performed. After removing the corresponding part of the *dura mater* (external meninge), the NO sensor was stereotaxically introduced into the internal medullar lamina of the thalamus (H = +5.5 mm; P = −3.14 mm/Bregma; L = +1.4mm), and then into the perifornical nucleus of the hypothalamus (H = +8.5mm; P = −3.14mm/Bregma; L = +1.4mm) according to Paxinos and Watson's atlas (4^th^ edition 1998) [14]. Reference (Ag/AgCl, 250 µm in diameter) and auxiliary (tungsten wire, 250 µm in diameter) electrodes were implanted in contact with external meninge. The differential NO oxidation current (650 mV vs Ag/AgCl) was measured with the instrumental set-up as already detailed [15]. The output signal intensity was recorded and quantified using a specially designed software (Saphir, Chapareillan, France). Measurements of stabilized signals were taken every 2 min. The experiments were performed in control and infected rats at D5, D10, D16, and D22 days post-infection (6 animals per experimental day of each group). For each infected animal, data were expressed as percentages of healthy controls. At the end of each experimental session, the position of the NO sensor was verified (current of 1 mA through the NO sensor). Brain was removed, frozen and sliced (coronal sections 20 µm thick) using a cryostat (Microcom France, Francheville, France). Brain sections were stained with cresol purple to confirm the correct position of the NO sensor.

#### NO measurements in blood

Immediately after sampling by direct puncture of the heart with a heparinized syringe, blood was introduced into an airtight gas chamber (0.4 mL) containing the NO sensor, reference and auxiliary electrodes as previously described [16]. Measurements were performed with the same instrumental set-up described previously [15].

### NOS Activity

NOS activity was measured by the conversion of [^3^H]L-arginine to [^3^H]L-citrulline according to the procedure described by Clément *et al.* [17]. For total NOS activity, 20 µL (about 100 µg of protein) of protein extracts from the cytosolic fraction (containing predominantly nNOS/iNOS) were incubated at 37°C for 20 min in buffer A with a final volume of 100 µL (HEPES 50 mM, pH 7.2 containing valine 50 mM, CaCl_2_ 1.25 mM, L-citrulline 1 mM, L-arginine 40 µM, L-ornithine 1 mM, NADPH 1 mM, EDTA 125 µM, (6R)-tetrahydrobiopterin 100 µM, FAD 5 µM, FMN 5 µM, calmodulin 1 µg, dithiothreitol 1 mM, and [^3^H]-L-arginine 87 nCi (41 Ci×mmol^−1^, Perkin Elmer Life Sciences, Courtaboeuf, France). Measurements of iNOS activity were performed in a buffer containing 5 mM EGTA as a calcium chelator. Blank values were obtained using buffer A plus NOS inhibitors, N^ω^-monomethyl-L-arginine (1 mM), N^ω^-nitro-L-arginine (10 mM), N^ω^nitro-L-arginine-methyl ester (10 mM) from Sigma-Aldrich and EGTA (5mM). The reaction was stopped by adding 2 mL of ice-cold buffer B (HEPES 20mM, EDTA 2mM, pH 5.5). Then, 1 mL of aqueous suspension of Dowex 50WX-8 resin (1/1 w/v), equilibrated with buffer B, was added. After agitation during 15 min, each tube was centrifuged for 3 min at 500 g and the supernatant was recovered. The resin was rinsed with 2 mL of distilled water. [^3^H]L-citrulline was quantified using a liquid scintillation analyzer (1900 TR, Packard Instrument, Rungis, France). Total NOS activity was calculated by subtracting blank values from the NOS assay. Activity of nNOS was estimated by subtracting iNOS activity value from total NOS activity value. All assays were made in duplicate and specific activity of NOS corresponding to picomoles of citrulline formed per min and per mg of protein (pmol/min/mg protein) was expressed as percentage of the values obtained in the non-infected condition. Peritoneal macrophages, collected from healthy rats treated or not by lipopolysaccharides (LPS), together with commercially available iNOS from mouse macrophages (50 U, CALBIOCHEM, France Biochem, Meudon, France), were used as iNOS activity control.

### Immunohistochemistry and Immunofluorescence

After anesthesia with chloral hydrate (400 mg/kg, i.p. injection), animals were perfused with Ringer lactate/heparin (0.05%) solution, followed by a cold mixture (PBS pH 7.4 containing paraformaldehyde at 4% and glutaraldehyde at 0.1%). Afterwards, brains were removed and immersed overnight at +4°C in a fixative mixture of PBS pH 7.4 containing 4% paraformaldehyde and then immersed for 3 days at +4°C in a sucrose solution at 30%. After being frozen in 2-methylbutane (Sigma-Aldrich) at −40 to −60°C, brain cryosections (25 µm thick) were obtained and collected in PBS containing 0.3% Triton X-100 (PBS-T) with 2% hydrogen peroxide then in PBS-T containing 0.01% sodium azide until use.

#### Immunostaining of iNOS and nNOS

As previously described [17], mouse anti-iNOS monoclonal antibody (1/10000, Sigma-Aldrich) or rabbit anti-nNOS polyclonal antibody (1/200, Santa Cruz Biotechnology, Santa Cruz, California, USA) were used as primary antibodies. Biotinylated horse anti-mouse (1/2000, Vector Labs, ABCYS, Paris, France) or biotinylated goat anti-rabbit (1/200, Vector Labs) were used as secondary antibodies. Peritoneal macrophages activated with LPS (0127-B8 and 0111-B4, 5µg/mL, Sigma-Aldrich) were used as positive iNOS controls 8 and 24 h after exposure to LPS.

#### Double labeling of iNOS with brain cell types: iNOS and neurons (iNOS/NeuN), iNOS and astrocytes (iNOS/GFAP), iNOS and microglia (iNOS/Integrin αM)

We used mouse anti-iNOS monoclonal antibody (1/10000, Sigma-Aldrich), mouse anti-NeuN antibody conjugated with Alexa Fluor 488 (anti-NEUronal Nuclei, Alexa Fluor 488 conjugated, 1/500, Millipore, Molsheim, France), rabbit anti-GFAP polyclonal antibody (anti-Glial Fibrillary Acidic Protein, 1/500, Dako laboratories, Carpentaria, California, USA) and mouse anti-Integrin αM (OX42) FITC conjugated antibody (1/500, Santa Cruz Biotechnology). As secondary antibodies, we used a goat anti-mouse antibody conjugated with Alexa Fluor 488 or 568 (1/500, Invitrogen- Life Technologies, Cergy-Pontoise , France) and a goat anti-rabbit antibody conjugated with Alexa Fluor 568 (1/500, Invitrogen). Visualization was achieved using a Vectashield mounting medium with DAPI (nuclear stain, Vector Labs) under a fluorescence microscope. Peritoneal macrophages activated with LPS (0127-B8 and 0111-B4, 5µg/mL, Sigma-Aldrich) during 24 h were used as positive iNOS controls.

#### Trypanosome labeling

Anti-*T. b. brucei* AnTat 1.1E from immunized mouse (1/400) and goat anti-mouse secondary antibody conjugated with Alexa Fluor 568 (1/500, Invitrogen) were used. Sera from non-infected mice were used as controls.

### Statistical Analysis

Results are expressed as mean ± SEM (n = 6 if not otherwise specified) of the different variables analyzed. Statistical analysis of differences between rat groups was performed using ANOVA. When ANOVA was significant at p<0.05, a post hoc Fisher's least significant differences test was applied.

## Results

### The Two-Stage Disease Course in *T. b. brucei*-Infected Rats

**Figure 1:**
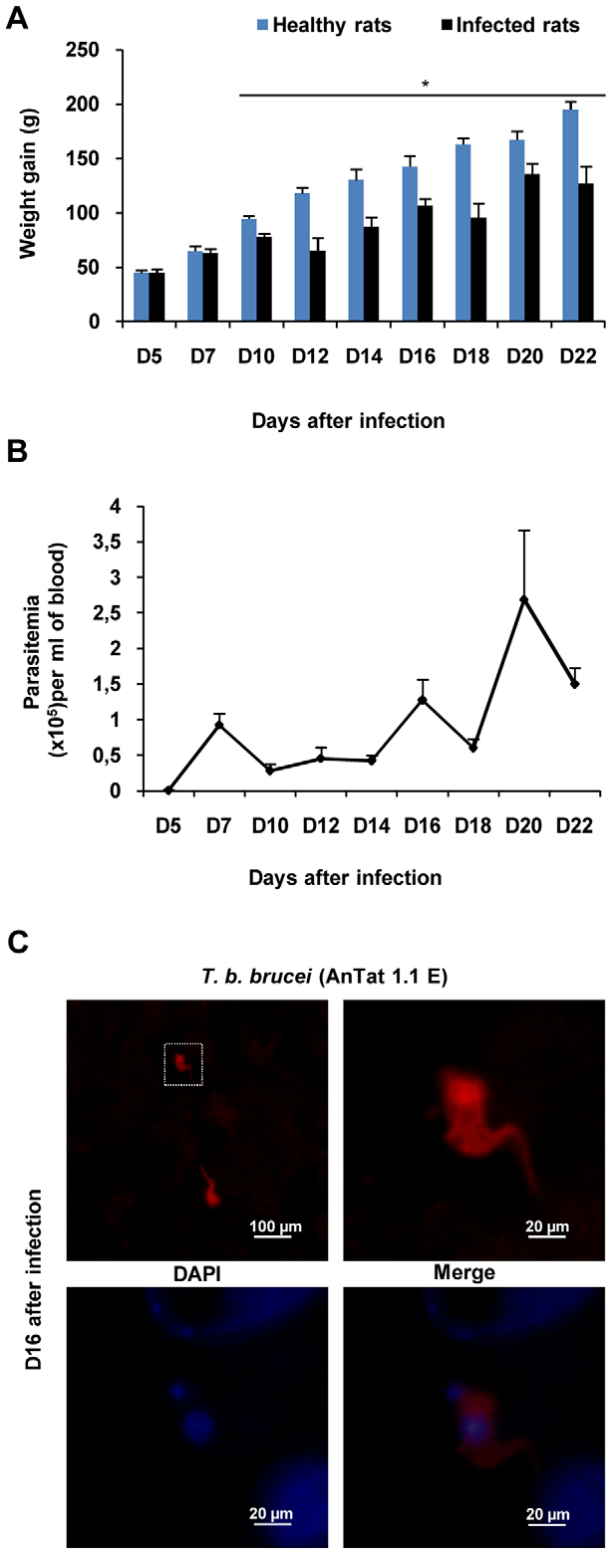
Biological diagnostic of experimental African trypanosomiasis in Wistar rats infected with *T. b. brucei*. (A) Body weight gain in healthy and infected rats calculated from weight values at D0 before infection: mean value±SEM (* p<0.05 compared to healthy rats, n = 6 per day and per group). (B) Time course of parasitaemia, n = 6 per day). (C) Immunofluorescent staining of *T. b. brucei* (red) in the brain parenchyma at D16 post infection (n = 3). Nuclei were stained with DAPI (blue). (Microscope Olympus BX51equipped with Olympus DP50 camera). Statistics analysis, ANOVA followed by post hoc Fisher's test. Abbreviations: *T. b. brucei*, *Trypanosoma brucei brucei*; DAPI, 4′, 6′ Di Amidino-2-Phenylindole.

Stage determination was evaluated by measuring body weight gain, trypanosome counts in the blood and CSF, and trypanosome immunostaining in the brain (from D0 to D22 post-infection). During the first week after infection, no difference in body weight gain was observed between control and infected rats. Control rats maintained a steady weight gain and a good health status (Fig. 1A). Infected animals experienced a significant body weight gain decrease from D10 (77.67±3.18 g *vs* 94.50±2.86 g in control rats) to D22 (126.83±15.63 g *vs* 195.20±7.32 g). Moreover, trypanosomes appeared in the blood five days after the intraperitoneal inoculation. As shown in Fig. 1B, parasitemia evolved in four successive waves occurring at intervals of 4–5 days with peak values at D7, D12, D16 and D22 post infection. The presence of trypanosomes in the CSF was detected at D10 post infection in 66% of the infected animals. At this time, parasites were not observed in the brain parenchyma. At D16 and D22 post infection, trypanosomes were found in the CSF and brain tissues (Fig. 1C) of all the animals (Table 1, n = 3 per day). The trypanosomes were observed mainly around the ventricular cavities (circum-ventricular organs).

**Table 1:** Presence of trypanosomes in the CSF of *T. b. brucei*-infected Wistar rats.

Days post-infection		D5	D10	D16	D22
		0	0	41	1
**Parasites number per µL** *	0	2	22	3	
		0	4	26	7

* Three infected animals per day.

*T. b. brucei* = *Trypanosoma brucei brucei*.

CSF = Cerebrospinal fluid.

### Opposite Changes Occurring in NO Synthesis between Brain and Blood

**Figure 2:**
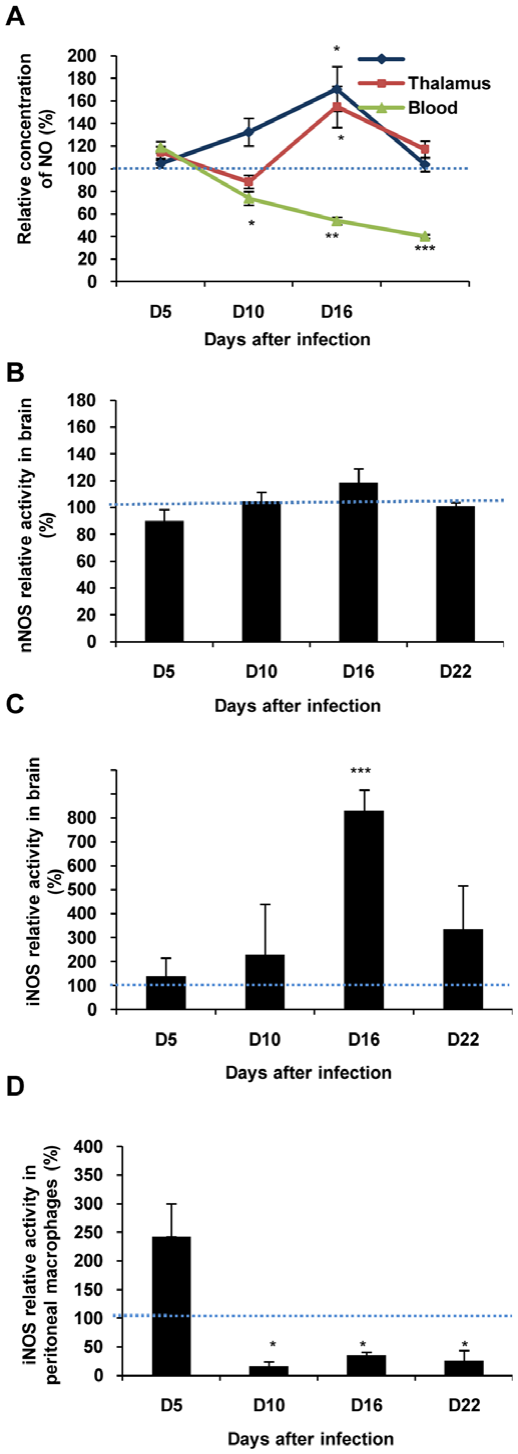
Changes occurring in NO synthesis in the brain and peripheral compartments of experimental African trypanosomiasis in Wistar rats infected with *T. b. brucei*. Variations in NO concentration or NOS activity (mean value ± SEM, n = 6 per day) were normalized with corresponding values measured in control rats (100%). (A) Relative concentration of NO analyzed in the blood, and in thalamic and hypothalamic areas. (B) Relative nNOS activity in thalamic and hypothalamic extracts. The 100% control value, delineated by the dotted line, corresponds to 337.8±34.5 pmol/min/mg. (C) Relative iNOS activity in thalamic and hypothalamic extracts. 100% correspond to 2.6±1.6 pmol/min/mg. (D) Relative iNOS activity in peritoneal macrophages. 100% correspond to 495.7±165.5 pmol/min/mg. Statistics analysis, ANOVA followed by post hoc Fisher's test. (*p<0.05, **p<0.001, ***p<0.0001 compared to healthy rats) Abbreviations: NO, nitric oxide; NOS, NO-synthase; iNOS, inducible NOS; nNOS, neuronal NOS.

In the brain, NO was measured successively in two different structures (hypothalamus and thalamus) in the course of the disease (from D5 to D22). As compared to control rats, infected animals exhibited a progressive increase in the extracellular concentration of NO. In the hypothalamus, the increase reached statistical significance at D16 (+71%, Fig. 2A). Afterwards, NO levels returned towards control values at D22. In the thalamic area, the changes were similar to those observed in the hypothalamus. After infection, the magnitude of NO concentration was maximal at D16 (+55% increase, Fig. 2A) compared to the value measured in non-infected control rats.

In the blood, a significant and progressive decrease in NO concentration was observed in infected rats (60% decrease between D5 and D22; Fig. 2A) as compared to the concentrations obtained in control animals.

### Cerebral and Peripheral Variations Occurring in NOS Activity

The NO production in the brain, may result from the activity of either nNOS or iNOS, while peripheral NO depends mainly on iNOS activity. In the hypothalamic and thalamic areas, no significant changes between control and infected animals were observed for nNOS activity in any examined slice between D5 and D22 post infection (Fig. 2B). On the contrary, infected animals experienced an important rise in iNOS activity from D10 to D16, the greatest elevation in enzyme activity occurring on D16 (up to +700% compared to controls). Afterwards, iNOS activity returned to control values on D22 (Fig. 2C).

In peritoneal macrophages, iNOS activity measures revealed opposite changes in comparison to brain data. An increase in the activity of the enzyme was observed in infected rats on D5 (+150% compared to non-infected controls). This variation was then followed by a drastic drop in the enzyme activity at D10 (−85% decrease compared with non-infected controls). Then, iNOS activity reached a plateau until D22 (−75% decrease) (Fig. 2D).

### Immunohistochemistry of iNOS and nNOS

**Figure 3:**
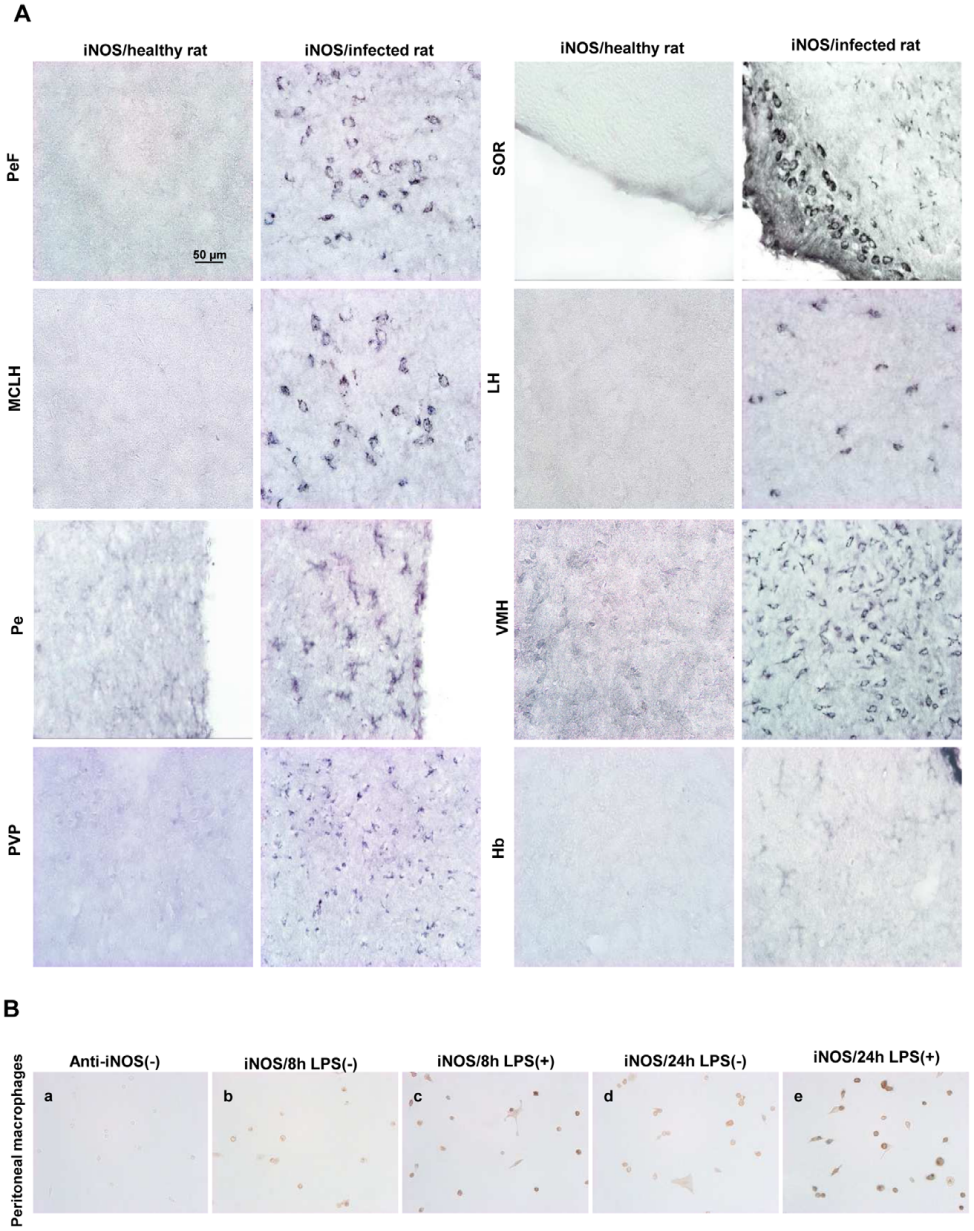
Immunostaining of iNOS in the brain of healthy and *T. b. brucei*-infected Wistar rats. (A) Immunohistochemistry for iNOS observed in different regions of the hypothalamus and thalamus. Perifornical nucleus (PeF); supraoptic nucleus; retrochiasmatic (SOR); magnocellular nucleus of the lateral hypothalamus (MCLH); lateral hypothalamic area (LH); periventricular hypothalamic nucleus (Pe); ventromedial hypothalamic nucleus (VMH); paraventricular thalamic nucleus, posterior (PVP); habenular nucleus (Hb). (B) Controls of iNOS labelling: peritoneal macrophages were incubated with (B-c, e) or without (B-b, d) LPS during 8 h (B-b, c) or 24 h (B-d, e). Specificity of secondary antibody was verified in incubating peritoneal macrophages without anti-iNOS antibody (B-a). Number of animals, n = 4 per group. The microscope used was an Olympus BX51 equipped with Olympus DP50 camera (objective×40). Abbreviations: iNOS, inducible NO-synthase; LPS, lipopolysaccharide.

In order to identify the cell types specifically involved in NO synthesis catalyzed by iNOS activity, an immunohistochemical study was performed in the sampled brain areas. With regard to iNOS, a cytosolic immunoreactivity was clearly evident at D16 in infected rats in several brain areas (pontine structures, cortical areas, amygdala, hippocampus, suprachiasmatic nuclei, Fig. 3A). With respect to the cell types involved, the hypothalamic iNOS immunoreactivity was located in neurons within the perifornical nucleus (PeF), supraoptic nucleus, retrochiasmatic (SOR), magnocellular nucleus of the lateral hypothalamus (MCLH), and lateral hypothalamic area (LH) structures [15], as well as in microglial cells within the periventricular hypothalamic nucleus (Pe). The iNOS labeling involved both cell types in the ventromedial hypothalamic nucleus (VMH). In the thalamus, the iNOS staining was particularly obvious around the dorsal part of the third ventricle (D3V) particularly in the paraventricular thalamic nucleus, posterior (PVP), and habenular nucleus (Hb) (Fig. 3A). The specificity of the anti-iNOS antibody employed was verified by an immunocytochemical investigation using LPS-treated or non-treated peritoneal macrophages. A loss of staining was observed when the anti-iNOS antibody was omitted in the immunocytochemical procedure (Fig. 3B a). However, most cells became strongly positive after LPS treatment (Fig. 3B c and e) as compared to untreated cells (Fig. 3B b and d). Concerning nNOS, although the immunostaining was clearly expressed in the brain, no significant differences were detected between non-infected controls and infected rats (data not shown).

### Immunofluorescence Identification of iNOS Expressing Cells

**Figure 4:**
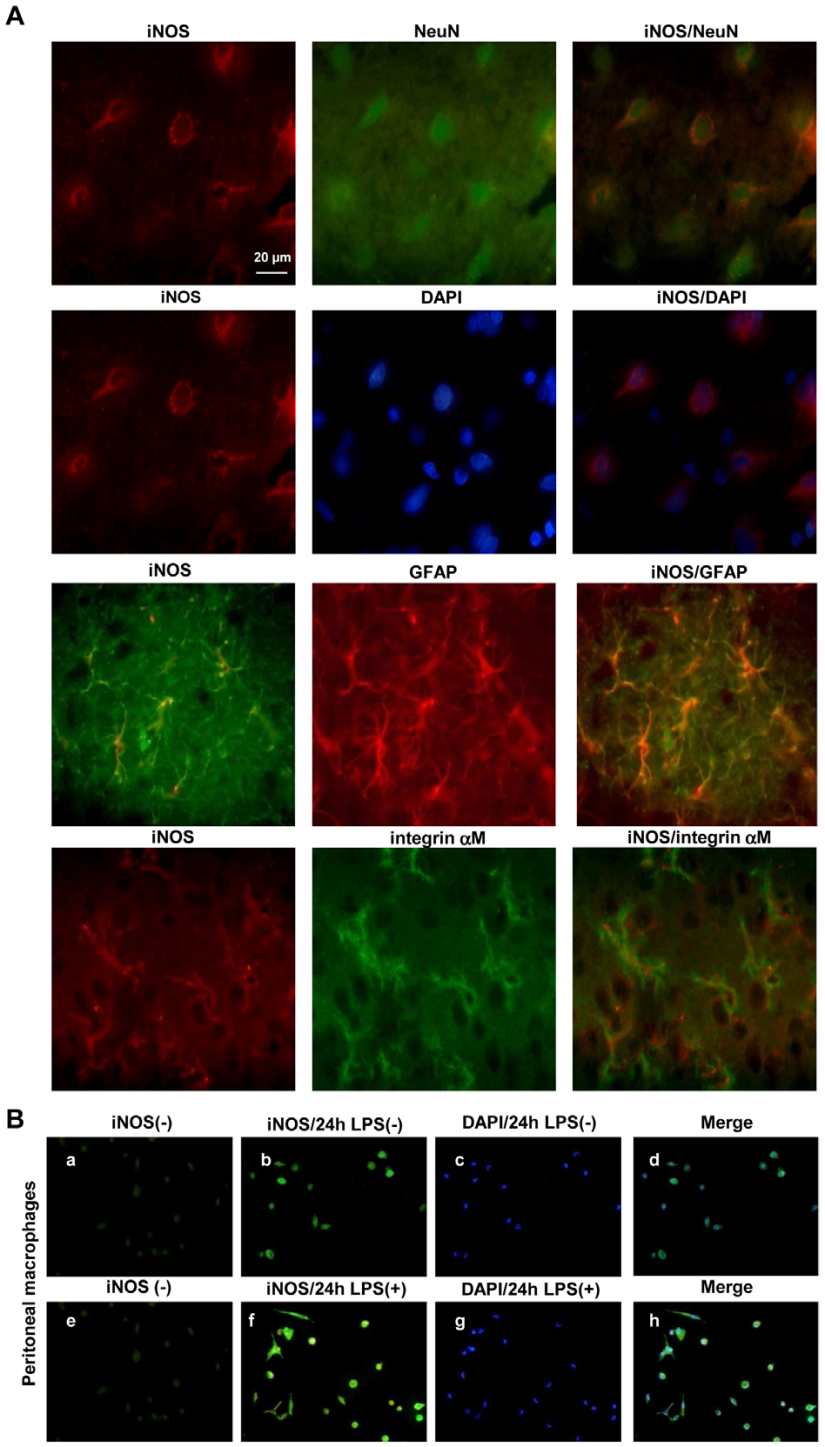
Localization of iNOS in neurons, astrocytes and microglia in *T. b. brucei*-infected rats. (A) The immunofluorescent staining of iNOS (red and green) was performed in the hypothalamic area. The different cell types in which iNOS was expressed were stained (double labelling) using NeuN (green) for neurons, GFAP (red) for astrocytes, and integrin αM (red) for microglia. (B) Controls of iNOS labelling: peritoneal macrophages were incubated with (B-f, g, h) or without (B-b, c, d) LPS during 24h. Specificity of secondary antibody was verified in incubating peritoneal macrophages without anti-iNOS antibody (B-a, e). Nuclei were stained with DAPI (B-c, g, blue). Number of animals, n = 4 per group. The microscope employed was an Olympus BX51 equipped with Olympus DP50 camera (objective×40). Abbreviations: iNOS, inducible NO-synthase; DAPI, 4′, 6′ Di Amidino-2-Phenylindole; NeuN, Neuron Nuclear; GFAP, Glial Fibrillary Acidic Protein; LPS, lipopolysaccharide.

Double iNOS/NeuN labeling (NeuN for neuronal cell bodies) and iNOS/DAPI labeling (DAPI for nuclei) revealed a clear cytosolic iNOS activity in neurons in infected rats. The iNOS activity was also present in astrocytes as shown by iNOS/GFAP (a specific marker for astrocytes) double labelling. When performing iNOS and Integrin αM double labelling (a specific marker for microglial cells), iNOS was also detected in microglial cells (Fig. 4A). The specificity of the immunofluorescence yielded by the iNOS was verified using peritoneal macrophages treated or not-treated with LPS during 24 h. A lack of immunofluorescence occurred in the macrophages that were not treated with LPS (Fig 4B).

## Discussion

In the present investigation, rats infected with *T. b. brucei* AnTat 1.1E were used as an experimental model for HAT. Our attention was especially focused on NO due to its trypanocidal properties [6]. Data reported here addressed: (i) the parameters necessary for a routine assessment of the two successive stages of the disease (body weight gain, parasitemia time course, CSF parasite counts), (ii) the changes in NO concentration occurring in the course of the neurological disease and the related NOS activity, as well as the identification of the cell types in which the cerebral NO-pathway changes take place.

### (i) Biological and Physiological Assessment of the Two Stages of the Disease

At D5 post-infection, the clinical signs characterizing the later meningoencephalitic phase were still absent. Weight gain did not differ between infected and control animals. Parasites appeared in blood but not in CSF samples. Considering the comparison with HAT, the infected animals were most certainly still in the hemolymphatic phase at D5 post-infection. At D10 and D16, the existence of a significant slowing in the rate of growth together with the presence of trypanosomes in the CSF indicated that the infected animals had entered the second or neurological stage of the disease. Furthermore, since body weight control resides at least partly in the hypothalamus, the decrease in growth progression observed might be considered as an early peripheral sign of neurological disturbances. In previous reports from our team [3], [18], the slowing in the rate of growth was considered to be characteristic of the second phase. It occurred a few days after the decrease in food intake and was associated with anemia and the infiltration of mononuclear inflammatory cells in the brain [3], [18]. We also demonstrated that trypanosomes can be present in the CSF in the absence of clinical symptoms [19]. We found that staining of trypanosomes in the brain was evidenced at D16, confirming previous observations done in the Sprague Dawley rat model [20]. Trypanosomes were observed around the ventricular cavities in areas where the BBB is most permeable, i.e., at the level of circumventricular organs, median eminence and subfornical organ of the hypothalamus [21]. Such localizations may contribute to disturbances observed in HAT (weight gain reduction [18], sleep and wake disturbances [2], endocrine modifications [2] and temperature deregulation [18]).

### (ii) Changes in the NO Production and Related Regulatory Processes

In the blood, the decrease in the NO fraction was evident mainly during the later stage of the disease, in agreement with previous observations from our team (mouse, rat, and human [9], [10]). These changes are likely to result from an impaired iNOS activity, which was found to occur in peritoneal macrophages collected from the same animals. Such a decrease in NO concentration has also been reported in mononuclear cells collected from the blood of *T. congolense*-infected cattle [22]. Moreover, a contribution of eNOS cannot be excluded, since an increase in eNOS activity has been reported in mice infected with *T. b. brucei* [23]. Even though such an effect exists, its magnitude would not be capable of counteracting the highly significant decrease observed in blood NO concentration.

The impaired blood NO production may result from an imbalance in the stimulatory/inhibitory elements of the immune system. The iNOS activity is up-regulated mainly by the pro-inflammatory cytokines IFN-γ, TNF-α and IL-2, and down-regulated by the immunosuppressive cytokines IL-10, IL-4 and TGF-β [24]. The latter three cytokines are recognized as very potent inhibitors of IFN-γ-stimulated monocyte-macrophage effectors function, including NO synthesis and microbicidal activity. During the early stage of the infection, secretion of pro-inflammatory cytokines (IFN-γ, TNF-α) and NO by macrophages provides the host protection by inhibiting parasitic development and helping the host to prolong specific immune defenses [25]. However, immunosuppressive cytokines such as IL-10, IL-4 and IL-13 (anti-inflammatory effect) are significantly elevated in the later stage of the infection [25], [26]. IL-10 inhibits specifically TNF-α, and its excessive production may contribute to trypanotolerance and immunosuppression [27]. However, the down-regulation exerted on the iNOS by IL-10 and other anti-inflammatory cytokines is not sufficient to explain the decreased NO production observed in the blood at the neurological stage of the infection. Anti-inflammatory interleukins such as IL-4 and IL-10 are inducers of arginase isoforms, although these cytokines are known to act on the expression of arginase I, but not that of arginase II [28]. Arginase activation may also result from a direct interaction of trypanosome products on cells of the immune system causing the production of polyamines, which are essential for trypanothion synthesis, and therefore for the growth and survival of trypanosomes [25]. NO production may therefore be reduced in macrophages through the competition of arginase with iNOS as both enzymes use the same substrate, L-arginine. This aminoacid is indeed decreased in the plasma of infected mice [29]. Furthermore, the circulatory decrease in NO might also be due to the competition between L-arginine and ADMA (asymmetric dimethylarginine), a potent endogenous inhibitor of NO synthesis [30].

In the brain, NO production increased and the higher values were observed at D16 after infection. Excessive NO production can lead to alterations of neuronal signaling and to cell damage through the cytotoxicity of NO oxidation derivatives. When NO is released in large quantities, it may combine with the superoxide anion (O^.−^_2_) to form peroxynitrite (ONOO^−^), a free radical brain tissue scavenger. Such an increase in NO production has been observed in the cortex of mice infected by *T. b. brucei* at D15 and D28 after infection [10]. In the present investigation, iNOS activity varied similarly and exhibited a marked increase at D16 after infection. This aspect is also confirmed by immunohistochemical data demonstrating iNOS immunoreactivity at NO measurement sites, noticeably in the thalamus and hypothalamus. These findings corroborate those obtained with NO voltammetric measurements and suggest that iNOS is involved in the overproduction of NO during the second stage of the infection. This view is further reinforced by the facts that in our experiments: (i) no significant variation was noticed in nNOS activity/protein and mRNA expression (data not shown); (ii) a specific iNOS inhibitor was capable of reducing the increase in NO production measured in the cortex of mice infected with *T. b. brucei* (data previously reported from our team [31]).

Moreover, in the posterior hypothalamus, iNOS immunoreactivity was found to be located in neurons particularly in the perifornical area as well as in the lateral part of the hypothalamus, where the hypocretin neurons implicated in sleep/wake regulation are located. These neurons play a crucial role in the maintenance of a consolidated period of wakefulness [32]. Their loss or a mutation of the gene encoding for hypocretin receptors are accompanied by narcoleptic attacks with SOREMPs (sleep-onset REM periods), in humans and animals [32]. Although SOREMPs are also observed in HAT, our group has demonstrated that they do not depend on a lack of hypocretin availability since the concentration of CSF hypocretin was found to be near normal control values in HAT patients [33]. The above aspects remain, nevertheless, to be further investigated.

Besides the neuronal expression, iNOS immunoreactivity was also observed in glial cells (astrocytes and microglia) at the second stage of the disease (part of the involved cellular elements also comes from infiltrating macrophages [34]). Astrocytes and microglia may also display such macrophage-related functions in relation with an iNOS-related production of NO. Other investigations reveal also that, *in vitro* and in presence of *T. b. brucei*, IFN-γ (produced in the brain by infiltrated CD8+ T cells [34]) stimulates the production of NO in astrocytes and microglia through the activation of iNOS at the transcriptional level [35].

In trypanosomiasis, the chronic inflammatory response can be induced by parasite components and/or autoimmune reactions, as molecular mimicries were found between neurological components and trypanosomes [27]. This could explain that, after the increase in cerebral NO observed at D16, NO production decreased at D22 post-infection. Two reasons may lead to this observation. It has been suggested that nitrate (an inert metabolite of NO) concentration in the brain of mice infected by *T. b. brucei* decreases in the late or final phase of CNS infection (*pre-mortem* phase with more severe inflammation) [36]. Such a decrease might reflect an extremely deleterious *pre-mortem* situation during which anti-inflammatory processes seem to be protecting the brain from severe inflammation such as observed in autoimmune encephalitis [37]. Recent studies suggest that inhibition of the kynurenine pathway in the late CNS stage of infection can reduce the severity of host inflammation response [38]. It may also simply reflect a *pre-mortem* general immunosuppressive syndrome [27].

#### Conclusion

Direct measurements of NO allowed an analysis of the mechanisms regulating NO production throughout the time-course of infection in a *T. b. brucei-*infected rat model of HAT. In the periphery (macrophages and blood), NO production decreased. This aspect is triggered by a complex cascade of events involving cytokines and most likely the arginase pathway. It allows the reduction of the trypanocidal pressure exerted by NO towards trypanosomes, thus constituting a potential target for the development of a new adapted pharmacotherapy. Conversely, the NO production increased in the brain. Such a deleterious rise in NO resulted essentially from the enhanced iNOS activity in neurons, astrocytes and microglial cells. It might serve as a marker of the deleterious inflammatory situation of the brain.
