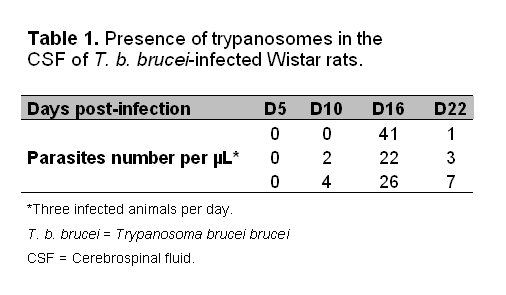# Correction: Cerebral and Peripheral Changes Occurring in Nitric Oxide (NO) Synthesis in a Rat Model of Sleeping Sickness: Identification of Brain iNOS Expressing Cells

**DOI:** 10.1371/annotation/98e97887-0f24-4113-9c24-0d4f4fe4ef77

**Published:** 2010-03-03

**Authors:** Donia Amrouni, Sabine Gautier-Sauvigné, Anne Meiller, Philippe Vincendeau, Bernard Bouteille, Alain Buguet, Raymond Cespuglio

In Table 1, the center row of data is misaligned. Please view the corrected Table 1 here: 

**Figure:**