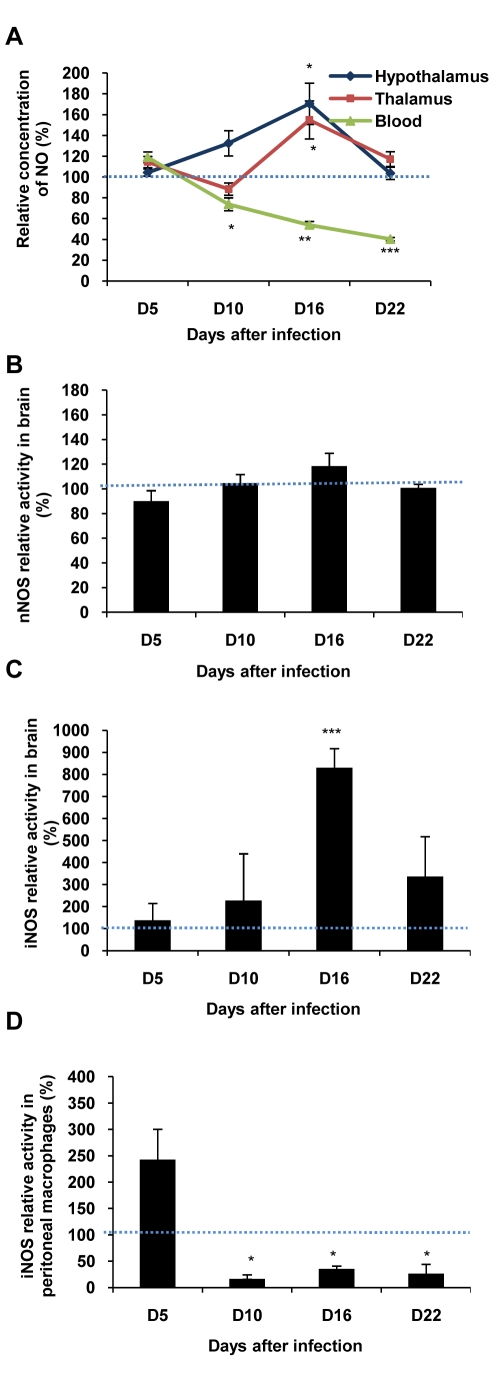# Correction: Cerebral and Peripheral Changes Occurring in Nitric Oxide (NO) Synthesis in a Rat Model of Sleeping Sickness: Identification of Brain iNOS Expressing Cells

**DOI:** 10.1371/annotation/07db26ac-e3b1-4c16-953a-dd06af38f621

**Published:** 2010-03-03

**Authors:** Donia Amrouni, Sabine Gautier-Sauvigné, Anne Meiller, Philippe Vincendeau, Bernard Bouteille, Alain Buguet, Raymond Cespuglio

In Figure 2A, the word "Hypothalamus" is missing from the key. Please view the corrected Figure 2 here: 

**Figure:**